# Bridging the gap between toxicity and carcinogenicity of mineral fibres by connecting the fibre crystal-chemical and physical parameters to the key characteristics of cancer

**DOI:** 10.1016/j.crtox.2021.01.005

**Published:** 2021-01-26

**Authors:** Alessandro F. Gualtieri

**Affiliations:** Department of Chemical and Geological Sciences, The University of Modena and Reggio Emilia, Modena, Italy

**Keywords:** Asbestos, FPTI, *In vitro* toxicity, Pathogenicity, IARC, mesothelioma, cancer

## Abstract

Airborne fibres and particularly asbestos represent hazards of great concern for human health because exposure to these peculiar particulates may cause malignancies such as lung cancer and mesothelioma. Currently, many researchers worldwide are focussed on fully understanding the patho-biological mechanisms leading to carcinogenesis prompted by pathogenic fibres. Along this line, the present work introduces a novel approach to correlate how and to what extent the physical/crystal-chemical and morphological parameters (including length, chemistry, biodurability, and surface properties) of mineral fibres cause major adverse effects with an emphasis on asbestos. The model described below conceptually attempts to bridge the gap between toxicity and carcinogenicity of mineral fibres and has several implications: 1) it provides a tool to measure the toxicity and pathogenic potential of asbestos minerals, allowing a quantitative rank of the different types (*e.g*. chrysotile vs. crocidolite); 2) it can predict the toxicity and pathogenicity of “unregulated” or unclassified fibres; 3) it reveals the parameters of a mineral fibre that are active in stimulating key characteristics of cancer, thus offering a strategy for developing specific cancer prevention strategies and therapies.

Chrysotile, crocidolite and fibrous glaucophane are described here as mineral fibres of interest.

## Highlights

• The model attempts to bridge toxicity and carcinogenicity of mineral fibres.
• The model measures the toxicity and pathogenic potential of asbestos minerals.
• The model predicts the toxicity and pathogenicity of unregulated/unclassified fibres.
• The model reveals the fibre parameters active as key characteristics of cancer.

## Graphical abstract

**Figure:**
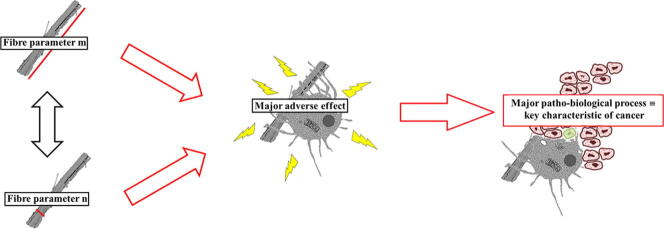

## 1 Introduction

Airborne particulates include fibrous particles of both natural and industrial origin. Undoubtedly, asbestos minerals are the most studied members of the realm of naturally occurring mineral fibres. The family of synthetic industrial fibres comprises glass/rock wool, carbon nanotubes and many more types (Gualtieri, 2012). The commercial term “asbestos” refers to six minerals, namely chrysotile (*i.e.,* the fibrous member of the serpentine group) and five fibrous amphiboles (*i.e.,* amphibole asbestos): actinolite asbestos, amosite (the fibrous variety of cummingtonite-grunerite), anthophyllite asbestos, crocidolite (the fibrous variety of riebeckite), and asbestos tremolite (Case et al., 2011, Gualtieri, 2017). Because of its unique technological properties, asbestos has been used in the human history since 3000BCE in the form of >3000 asbestos-containing materials (ACM) (Gualtieri, 2017a). The overwhelming industrial age of asbestos began in the second half of the 19^th^ century when mine mechanization promoted the exploitation of large chrysotile deposits in Canada (e.g., Lake Asbestos of Quebec), Russia and Europe (e.g., the Balangero mine, Italy) and amphibole asbestos mines in South Africa (Ross and Nolan, 2003).

Although there was early sporadic evidence of asbestos disease at the beginning of the 20^th^ century (Case and Marinaccio, 2017), after World War II an increasing number of scientific studies revealed that exposure to asbestos fibres in the working environment was associated with fatal diseases such as lung cancer. The mile stone in this regard was the cohort study of Sir Richard Doll who unequivocally demonstrated the link between lung cancer and exposure to asbestos fibres (Doll, 1955). Later, numerous cohort and case control studies indisputably demonstrated the association between asbestos exposure and lung diseases like carcinoma, malignant mesothelioma (MM), asbestosis and others (IARC, 2012). For this reason, the International Agency for Research on Cancer (IARC) classified in 1989 all the six forms of asbestos as *Group 1* “substances carcinogenic to humans” (IARC, 2012).

Sixty-five years after the pioneering work of Sir Richard Doll, we have learned much about the biological processes that promote toxicity and pathogenicity of mineral fibres. In this paper, the following definitions of toxicity and carcinogenicity (Mossman et al., 2011) are used: toxicity intended as “genotoxicity” is the property of an agent for altering the genome of cells resulting in cell death or altered function and division of cells, including alterations in the genetic material; toxicity intended as “mutagenicity” is the property of an agent to cause alterations in the genome that are transferred to cell progeny and subsequent generations; carcinogenicity is the property of an agent to alter the genome and/or cellular control processes resulting in uncontrolled cell proliferation and other functional or phenotypic changes that in turn result in malignant cancers.

It is now universally accepted that, to a first approximation, long, thin, biodurable asbestos fibres reach the alveolar space and pleural/peritoneal surface where they induce chronic inflammation and adverse effects, responsible for the onset of lung cancer, MM and other lung diseases (Stanton et al., 1981, Lentz et al., 2003, Donaldson et al., 2010, Lippmann, 2014, Carbone et al., 2019, Wylie et al., 2020). We know now that asbestos-related carcinogenesis is the result of a complex multistep process governed by the interplay of all the structural and physical/chemical characteristics of fibres: morphology (e.g.*,* length and width; Donaldson et al., 2010); chemical composition (e.g.*,* iron and heavy metals content; Fubini and Mollo, 1995, Gualtieri et al., 2019a); surface activity (e.g.*,* zeta potential and cation exchange capacity; Pollastri et al., 2014); biodurability (*i.e.,* the resistance of fibres to chemical/biochemical alteration: Bernstein et al., 2005, Gualtieri et al., 2018a, Gualtieri et al., 2019b). Because of this complexity, there are still open issues and disputes on the mode of action *in vivo* and the effective toxicity and pathogenicity of mineral fibres (Gualtieri et al., 2017). The most critical dispute regards the so-called “global chrysotile issue”. Sixty-seven countries worldwide have banned all six asbestos minerals (International Ban Asbestos Secretariat, 2020), the others, including China, India, Kazakhstan, and Russia still allow “safe use” (*i.e.*, its controlled manipulation wearing individual protection devices) of chrysotile. This model assumes that, compared to amphibole asbestos, chrysotile is less potent for the induction of MM and low exposures to chrysotile do not present a detectable risk to health (Roggli, 1995, Hodgson and Darnton, 2000, Garabrant and Pastula, 2018, Roggli and Vollmer, 2008, Mossman et al., 2011, Bernstein et al., 2013). This model is supported by the different biopersistence of chrysotile with respect to amphibole asbestos. Chrysotile is not biodurable and easily leached *in vivo* in the lungs whereas amphibole asbestos fibres are biodurable (Jaurand et al., 1977, Morgan, 1994, Bernstein et al., 2013) and induce chronic inflammation responsible for adverse effects. However, members of the scientific community and regulatory agencies support the model that all asbestos minerals are toxic and pathogenic and all increase the risk of MM given that biodurability alone cannot explain the toxicity and pathogenicity of asbestos (Collegium Ramazzini, 2010). To the eye of the author, a way to reach a universal consensus on the “global chrysotile issue” and all the other open issues on the toxicity/pathogenicity of mineral fibres is to revise and rationalize at a basic level the relationship between the various fibre parameters and the patho-biological processes *in vivo* responsible for the induction of cancer. It should be clearly assessed if and to which extent a mineral fibre is toxic, if the mineral fibre is carcinogenic and how toxicity and pathogenicity are related.

A working group of IARC recently reviewed all information and data of human carcinogenesis mechanisms and found that *Group 1* agents (like asbestos minerals) commonly show one or more of 10 key characteristics that distinguish them as carcinogenic to humans (Smith et al., 2016, Krewski et al., 2019). The 10 key characteristics of carcinogens are listed in Table 1. Alongside, a model to quantitatively assess the toxicity/pathogenicity potential of mineral fibres has been proposed (Gualtieri, 2017, Gualtieri, 2018, Mossman and Gualtieri, 2020). It delivers a Fibre Potential Toxicity/Pathogenicity Index (FPTI) based on all physical/crystal/chemical and other parameters (Table 2) that induce biological mechanisms responsible for their adverse effects (Gualtieri, 2018). This work describes a comprehensive model that fills the gap between the two worlds, with an original approach aimed at linking each physical/crystal-chemical and morphological parameters of a fibre to the major adverse effect that it causes *in vivo* and, in turn, to the patho-biological process classified as key characteristic of cancer. For example, a long biodurable asbestos fibre (parameter (1,1) in Table 2) prompts frustrated phagocytosis of macrophages (major adverse effect) that in turn leads to oxidative stress and chronic inflammation (major patho-biological processes) classified as key characteristic of cancer nr. (5.) and (6.) in Table 1. Cross-correlations must be considered because chronic inflammation is due to both the length of the fibres and their biodurability (parameters (1,1) and (1,12), respectively, in Table 2).

**Table 1:** IARC 10 Key characteristics (pathological processes) exhibited by agents known to cause cancer in humans (adapted from Smith et al., 2016).

1.	Electrophilicity
2.	Genotoxicity
3.	Alteration of DNA repair or genomic instability
4.	Epigenetic alteration
5.	Oxidative stress
6.	Chronic inflammation
7.	Immunosuppression
8.	Modulation of receptor-mediated effects
9.	Immortalization
10.	Alteration of cell cycle and especially changes in growth factors and signalling pathways

**Table 2:** Physical/chemical and morphological parameters of the fibre potential toxicity index (FPTI) model to predict *ab initio* the toxicity/pathogenicity of minerals fibres (from Gualtieri, 2018).

Parameter	Code*
Morphometry	
length	(1,1)
width	(1,2)
crystal curvature	(1,3)
crystal habit	(1,4)
fibre density	(1,5)
hydrophobic character of the surface	(1,6)
surface area	(1,7)

Chemistry	
Total iron content	(1,8)
ferrous iron	(1,9)
Surface ferrous iron/iron nuclearity	(1,10)
content of metals other than iron	(1,11)

Biodurability	
dissolution rate	(1,12)
velocity of iron release	(1,13)
velocity of silica dissolution	(1,14)
velocity of release of metals	(1,15)

Surface activity	
zeta potential	(1,16)
fibres’ aggregation	(1,17)
cation exchange in zeolites	(1,18)

*Parameters are labelled using a matrix-like notation. As explained in Gualtieri (2018), each parameter is a row element of a symmetric m × m matrix with (1,1) = (2,1), (1,2) = (2,2) … so that each element may correlate with the others.

The model that has been developed is intended to be a basic paradigm which simply predicts if a unit (a statistically representative sample of individuals characterized by a length *x*, width *y*, specific surface area *w* …) of a mineral fibre possesses a toxicity/pathogenicity potential when inhaled and hosted in the lung environment, and quantitatively compares its potential to that of other mineral fibres. The adverse effects prompted by the fibre *in vivo* leading to toxic/pathogenic mechanisms do not refer to any specific lung disease. In this regard, the model is independent on both dose (following the original concept of Paracelsus) and time (following the original concept of Haber) toxicity parameters and cancer dose–response relationship. To make an example, this basic model predicts that crocidolite has a high FPTI (high potential to prompt adverse effects in the lungs responsible for cyto-toxic, geno-toxic and pathogenic mechanisms) and that fibrous sepiolite has a negligible FPTI but it does not tell if a very high cumulative dose of fibrous sepiolite leads to lung fibrosis (asbestosis) while a very low cumulative dose of crocidolite does not. The FPTI model has been applied to a representative suite of mineral fibres like chrysotiles, amphiboles and zeolites (Gualtieri, 2018)

This basic approach has several implications. A clear picture of the relationship between the known fibre parameters and the key hallmarks of lung cancers helps in properly weighing the toxic and pathogenic potential of asbestos and mineral fibres other than asbestos. Because each fibre parameter is considered, it is possible to assess if and how many key characteristics of cancer are induced by that parameter. This is of paramount importance in knowing the specific key characteristics of cancer that are turned on by a fibre species. Moreover, this information can help in developing targeted prevention strategies and therapies, given that the nature of the fibre the patient was exposed to is known (no matter if the exposure occurred during his professional activity or environmental). For example, the target therapies for MM patients should be very different if the asbestos fibres that caused exposure were iron-free or iron-rich (Gualtieri et al., 2019a). Specifically, in the latter case, phlebotomy and iron chelation therapies, although not yet universally accepted, may be eventually successful in the prevention of MM (see Toyokuni, 2019). Another implication of this tool is to predict the toxicity and pathogenicity of “unregulated” or unclassified minerals or industrial fibres. In nature a large number of “unclassified” minerals fibres share some of the characteristic of asbestos and therefore their potential adverse effects (Carbone et al., 2016). Among them are fibrous glaucophane (Di Giuseppe et al., 2019), fibrous ferrierite (Gualtieri et al., 2018b, Zoboli et al., 2019), fibrous offretite (Mattioli et al., 2018) and many more. Recently, the risk posed by unregulated fibrous amphiboles (e.g.*,* winchite and richterite) has been indicated by several studies (Baumann et al., 2015, Naik et al., 2017) and although these fibres have not yet been classified by the IARC, specific *in vitro* and *in vivo* tests have assessed that they represent a potential hazard for human health (Baumann et al., 2015, Naik et al., 2017).

## 2 The basic FPTI model

The FPTI model is described in detail in Gualtieri (2018) and Mossman and Gualtieri (2020). There are 18 morphological, crystal-chemical and physical parameters used to describe a mineral fibre as a whole. Among them, the length (1,1) and width (1,2) are key factors in toxicity, inflammation and pathogenicity (Stanton et al., 1981, Donaldson et al., 2010). The curvature (1,3) of the fibre surface affects the binding process of proteins and influences cell adhesion (Churg, 1993) while the crystal habit (1,4) and the density (1,5) influence the depositional pathway of the fibre in the respiratory tract (Gualtieri, 2018). The hydrophobic character of a fibre (1,6) rules the interaction with biopolymers (*i.e.*, proteins) and phagocytic cells (Gualtieri et al., 2017, Gualtieri, 2018) while the surface area (1,7) affects the dissolution kinetics, biodurability, and resistance to chemical/biochemical alteration (Donaldson et al., 2010, Gualtieri et al., 2018a).

Among the chemical parameters, iron content related parameters (1,8) and (1,9) are involved in the direct formation of reactive oxygen species (ROS). Iron (mainly Fe^+2^) at the surface of asbestos fibres, or released by them in the intracellular space, promotes the formation of hydrogen peroxide (H_2_O_2_) and hydroxyl radicals (HO•) via the Haber-Weiss cycle [1] and the Fenton reaction [2].

$$\left[1\right]{\mathrm{Fe}}^{2+}+O_{2}\to {\mathrm{Fe}}^{3+}+O_{2}^{.-}$$

$$O_{2}^{.-}+2H^{+}+e^{-}+H^{+}\to H_{2}O_{2}$$

$$\left[2\right]{\mathrm{Fe}}^{2+}+H_{2}O_{2}\to {\mathrm{Fe}}^{+3}+{\mathrm{OH}}^{-}+{\mathrm{HO}}^{.}$$

ROS generation (e.g.*,* peroxides, superoxide and hydroxyl radical), overwhelming the antioxidant cell defence, induces alteration of membrane lipids and proteins, cell injury and DNA damage (Wang et al., 2017, Mossman, 2018). Reactivity of iron is also related to its nuclearity (1,10) (Gualtieri, 2018). Metals other than iron like chromium, nickel, manganese and others (1,11) may prompt inflammation *in vivo* and production of ROS.

The biodurability-related parameters are based on the dissolution rate of the fibre (1,12). It is assumed that if a fibre rapidly dissolves in lung fluids (*i.e.*, it has a low biodurability), it is not biopersistent and in principle is less toxic than a fibre with high biodurability (Bernstein et al., 2005; 2013). The rate of dissolution of iron (1,13), silica (1,14) and metals (1,15) in the extracellular space may also in principle generate ROS. The way silica induces production of ROS is deemed to be governed by surface silanol density (Zhang et al., 2012) but is still a matter of debate.

As far as the surface activity is concerned, zeta potential (1,16) influences a number of phenomena responsible for adverse effects and the agglomeration of the fibres (1,17) (Light and Wei, 1977, Pollastri et al., 2014). The last parameter (1,18) is the cation exchange prompted by zeolite fibres whose influence on the bio-chemical processes *in vivo* is not well understood yet.

For each parameter, a weighed score is assigned. The weighing scheme includes cross-correlations of the parameters (Gualtieri, 2018, Mossman and Gualtieri, 2020) based on their step/hierarchy H where w_1_ = 1/H with H = 1 = not correlated, 2 = correlated or 3 = strongly correlated. A weight defined as w_2_ = 1/U is also applied to each parameter and accounts for the uncertainty in its determination. It is defined by the penalty parameter U with 1 = low to null uncertainty, 2 = some degree of uncertainty, 3 = high uncertainty. FPTI_i_ is then defined as (Gualtieri, 2018):

$${\text{FPTI}}_{\text{i}}=\sum_{\text{i}=\text{1}}^{\text{n}}{\text{w}}_{\text{1}}\cdot {\text{w}}_{\text{2}}\cdot {\text{T}}_{\text{i}}$$

with T_i_ = class value of the parameter i of the model; w_1_ = 1/H weight of the parameter according to its hierarchy H; w_2_ = 1/U weight of the parameter according to the uncertainty U of its determination.

## 3 The FPTI model linked to the key characteristics of carcinogens

**Fig. 1:**
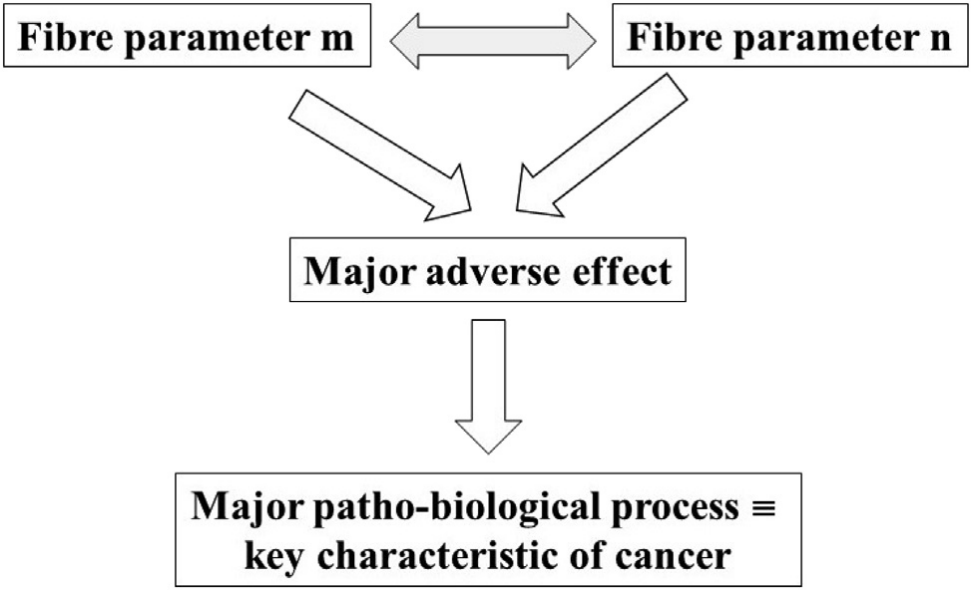
Flow chart of the model that relates the physical/crystal-chemical and morphological parameters of mineral fibres to the major adverse effect they prompt *in vivo* and, in turn, to the patho-biological processes known as key characteristic of cancer that are switched on. Possible cross-correlations between single fibre parameters are also show in grey.

Fig. 1 is a summary flow chart showing the logic beyond the model developed to link the 18 physical/crystal-chemical and morphological parameters of mineral fibres to the major adverse effects they prompt *in vivo* and, in turn, to the patho-biological processes linked to the 10 key characteristics of cancer (Table 1). The basic deductive logic used to design Table 3 is: if a parameter of a fibre (column 1) provokes a major adverse effect (column 2) responsible for a patho-biological process classified as a key characteristic of cancer (column 3), then the fibre possesses that specific key characteristic of cancer. Electrophilicity (1.), the first key characteristic of cancer, is chosen to explain the rationale beyond the model. In this case, it should be assessed if the fibre is electrophilic. In other terms, does the fibre possesses parameters that directly or indirectly make it electrophilic? Long (1,1) fibres that cannot be easily engulfed by alveolar macrophages (AM) prompt frustrated phagocytosis and indirectly the production of electrophilic species like ROS. Surface ferrous iron (1,10) is a chemical parameter that directly prompts the production of ROS at the surface of the fibre both *in vitro* and *in vivo*. In contrast, if a fibre is short and engulfed by AM, it does not prompt frustrated phagocytosis and indirect production of ROS. If it’s iron-free, it will not prompt surface-mediated production of ROS *in vivo*. Table 3 also shows that each parameter depends upon others. For example, the fibre length (1,1) depends upon the fibre dissolution rate (1,12) regulating the length of the fibre with time *in vivo* and therefore the triggering of frustrated phagocytosis. Evident cross-correlations are also found for chemical parameters like the content of iron (1,8) or metals (1,13) with their dissolution rates (1,13) and (1,15), respectively.

**Table 3:** Key characteristics/pathological process known to cause cancer in humans. For each patho-biological process featuring the 10 IARC key characteristics (Smith et al., 2016), the major adverse effects induced by specific fibre’ parameters (see the list in Table 1) are reported.

Fibre parameter	Major adverse effect	Key characteristic of carcinogenicity (patho-biological process)
length (1,1)	Prompts indirect production of electrophilic species like hydroxyl radicals (ROS) due to alveolar macrophages (AM) frustrated phagocytosis	1. electrophilicity
surface area (1,7)	Rules the overall size of the fibre *in vivo* with indirect production of ROS if the fibre is long enough to cause frustrated phagocytosis	
total iron content (1,8) ferrous iron (1,9) surface ferrous iron (1,10) content of metals other than iron (1,11)	Prompt direct production of electrophilic species like hydroxyl radicals ROS by metal-mediated Fenton type reaction at the fibre’ surface	
dissolution rate (1,12)	Rules the length of the fibre *in vivo* with indirect production of ROS if the fibre is long enough to cause frustrated phagocytosis	
velocity of iron release (1,13) velocity of silica release/formation (1,14) velocity of release of metals (1,15)	Rule the rate of (direct) production of ROS at the fibre’ surface or at the surface of newly-formed silica relicts (e.g. after dissolution of chrysotile: Gualtieri et al., 2019c)	
---------------------	------------------------------------------------	--------------------------------
length (1,1)	Prompts indirect production of genotoxic ROS/RNS (reactive nitrogen species) during AM frustrated phagocytosis	2. genotoxicity
surface area (1,7)	Rules the overall size of the fibre *in vivo* with indirect production of genotoxic ROS/RNS if the fibre is long enough to cause frustrated phagocytosis	
total iron content (1,8) ferrous iron (1,9) surface ferrous iron (1,10) content of metals other than iron (1,11)	Prompt direct production of genotoxic ROS by metal-mediated Fenton type reaction at the fibre’ surface	
dissolution rate (1,12)	Rules the length of the fibre *in vivo* with indirect production of genotoxic ROS/RNS if the fibre is long enough to cause frustrated phagocytosis	
velocity of iron release (1,13) velocity of silica release/formation (1,14) velocity of release of metals (1,15)	Rule the rate of (direct) production of genotoxic ROS/RNS at the fibre’ surface or at the surface of newly-formed silica metastable products	
zeta potential (1,16)	Rules the production of genotoxic ROS/RNS at the fibre’ surface	
---------------------	------------------------------------------------	--------------------------------
length (1,1)	Prompts AM-induced frustrated phagocytosis causing indirect production of ROS/RNS responsible for alteration of DNA repair and chromosomic instability/defectivity	3. alteration of DNA repair or genomic instability
surface area (1,7)	Rules the overall size of the fibre *in vivo* with indirect production of ROS/RNS	
total iron content (1,8) ferrous iron (1,9) surface ferrous iron (1,10) content of metals other than iron (1,11)	Prompt direct production of ROS/RNS at the fibre’ surface	
dissolution rate (1,12)	Rules the length of the fibre *in vivo* with indirect production of ROS/RNS during AM frustrated phagocytosis	
velocity of iron release (1,13) velocity of release of metals (1,15)	Rule the rate of (direct) production of ROS/RNS at the fibre’ surface	
zeta potential (1,16)	Rules the production of ROS/RNS at the fibre’ surface	
---------------------	------------------------------------------------	--------------------------------
length (1,1)	Prompts AM-induced frustrated phagocytosis causing indirect production of ROS/RNS responsible for epigenetic alteration	4. epigenetic alteration
surface area (1,7)	Rules the overall size of the fibre *in vivo* with indirect production of ROS/RNS	
total iron content (1,8) ferrous iron (1,9) surface ferrous iron (1,10) content of metals other than iron (1,11)	Prompt direct production of ROS/RNS at the fibre’ surface	
dissolution rate (1,12)	Rules the length of the fibre *in vivo* with indirect production of ROS/RNS during AM frustrated phagocytosis	
velocity of iron release (1,13) velocity of release of metals (1,15)	Rule the rate of (direct) production of ROS/RNS at the fibre’ surface	
zeta potential (1,16)	Rules the production of ROS/RNS at the fibre’ surface	
---------------------	------------------------------------------------	--------------------------------
length (1,1)	Prompts indirect production of genotoxic ROS during AM frustrated phagocytosis causing the oxidative burst	5. oxidative stress
surface area (1,7)	Rules the overall size of the fibre with indirect production of ROS due to frustrated phagocytosis	
total iron content (1,8) ferrous iron (1,9) surface ferrous iron (1,10) content of metals other than iron (1,11)	Prompt direct production of ROS by metal-mediated Fenton type reaction at the fibre’ surface	
dissolution rate (1,12)	Rules the overall size of the fibre with indirect production of ROS due to frustrated phagocytosis	
velocity of iron release (1,13) velocity of silica release/formation (1,14) velocity of release of metals (1,15)	Rule the rate of production of ROS at the fibre’ surface or at the surface of newly-formed silica metastable products	
zeta potential (1,16)	Rules the production of ROS at the fibre’ surface	
---------------------	------------------------------------------------	--------------------------------
length (1,1)	Prompts local chronic inflammation due to AM frustrated phagocytosis	6. chronic inflammation
hydrophobic character of the surface (1,6)	Influences cell uptake (Gualtieri et al., 2017) and consequently the viability of AM phagocytosis and local chronic inflammation	
surface area (1,7)	Rules the overall size of the fibre *in vivo* and consequently AM frustrated phagocytosis and local chronic inflammation	
total iron content (1,8) ferrous iron (1,9) surface ferrous iron (1,10) content of metals other than iron (1,11)	Prompt direct production of ROS/RNS, cause of local chronic inflammation (Robinson and Coussens, 2005)	
dissolution rate (1,12)	Determines the persistence at site of deposition, triggering chronic inflammatory activity	
velocity of iron release (1,13) velocity of release of metals (1,15)	Rule the rate of direct production of ROS/RNS causing local chronic inflammation	
zeta potential (1,16)	Rules the production of ROS/RNS at the fibre’ surface causing local chronic inflammation	
fibres’ aggregation (1,17)	Rules the aggregation of fibres *in vivo*, playing a role in AM frustrated phagocytosis. Aggregates are less prone to be successfully engulfed by AM (Gualtieri et al., 2017) and cause local chronic inflammation	
---------------------	------------------------------------------------	--------------------------------
length (1,1)	Prompts local chronic inflammation/immunosuppression due to AM frustrated phagocytosis	7. immunosuppression
hydrophobic character of the surface (1,6)	Influences cell uptake, AM phagocytosis and local chronic inflammation/immunosuppression	
surface area (1,7)	Rules the overall size of the fibre *in vivo* and consequently AM frustrated phagocytosis and local chronic inflammation/immunosuppression	
total iron content (1,8) ferrous iron (1,9) surface ferrous iron (1,10) content of metals other than iron (1,11)	Prompt direct production of ROS/RNS, cause of local chronic inflammation/immunosuppression	
dissolution rate (1,12)	Determines the persistence at site of deposition, triggering chronic inflammatory activity	
velocity of iron release (1,13) velocity of release of metals (1,15)	Rule the rate of direct production of ROS/RNS causing local chronic inflammation/immunosuppression	
zeta potential (1,16)	Rules the production of ROS/RNS at the fibre’ surface local chronic inflammation/immunosuppression	
fibres’ aggregation (1,17)	Rules the aggregation of fibres *in vivo* and AM frustrated phagocytosis, causing local chronic inflammation/immunosuppression	
---------------------	------------------------------------------------	--------------------------------
length (1,1) width (1,2) crystal curvature (1,3) hydrophobic character of the surface (1,6)	Rules the nature and strength of the surface interaction with the cells	8. modulation of receptor-mediated effects
total iron content (1,8) ferrous iron (1,9) surface ferrous iron (1,10) content of metals other than iron (1,11)	Direct interaction of the fibres with cell surface and production of ROS/RNS at the fibre surface	
velocity of iron release (1,13) velocity of release of metals (1,15)	Rule the rate of direct production of ROS/RNS at the fibre surface	
---------------------	------------------------------------------------	--------------------------------
–	There are no literature data showing asbestos-induced disruption of specific cellular pathways to promote aberrant replication like various DNA and RNA viruses do	9. immortalization
---------------------	------------------------------------------------	--------------------------------
length (1,1) width (1,2) crystal curvature (1,3) hydrophobic character of the surface (1,6)	Rule the nature and strength of the surface interaction with the cells	10. alteration of cell cycle and especially changes in growth factors and signalling pathways
total iron content (1,8) ferrous iron (1,9) surface ferrous iron (1,10) content of metals other than iron (1,11)	Direct interaction of the fibres with cell surface and production of ROS/RNS at the fibre surface	
velocity of iron release (1,13) velocity of release of metals (1,15)	Rule the rate of direct production of ROS/RNS at the fibre surface	
cation exchange in zeolites (1,18)	It may affect/disrupt the calcium, sodium and potassium mediated intracellular signalling pathways and cross talk	

The characteristics of the fibre prompting major adverse effects related to genotoxicity (2.) are also responsible for the effects causing alteration of DNA repair or genomic instability (3.) and epigenetic changes (4.). In fact, alteration of DNA repair or genomic instability may result from both indirect effects of ROS and RNS and a direct physiological interaction between the cell and the fibre (Mossman et al., 1997, Nymark et al., 2008, Toumpanakis and Theocharis, 2011). Although DNA damage-inducible genes, such as TP53 and GADD153, are up-regulated in asbestos treated cells (Johnson and Jaramillo, 1997), accumulation of fibres in the lung and the continuous production of ROS/RNS causes repeated DNA damage, leading to increased genetic instability, a hallmark of neoplastic development (Nymark et al., 2008). Although the mechanism by which asbestos induces epigenetic changes is not fully understood, several studies have shown that both phagocytosis of fibres by AM and oxido-reduction reactions on fibre surfaces are known to generate ROS that result in DNA damage and oxidative stress, leading to genetic alterations (Cheng et al., 2020). High iron content of amphibole asbestos explains how iron-induced Fenton reactions also contribute to increased ROS (Cheng et al., 2020) and it was observed that iron-rich amphibole asbestos species induce phosphorylated histone γ-H2AX (Msiska et al., 2010) marker of epigenetic alteration and gene-specific DNA methylation (Mossman, 2017, Öner et al., 2018, Mossman and Gualtieri, 2020).

Oxidative stress (5.) is a key characteristic of cancer produced by an imbalance between production of free radicals and ROS, and their elimination by protective mechanisms, referred to as antioxidants (Reuter et al., 2010). It is caused by ROS generated by the chemical elements present at the surface of the fibres or by mechanical damage induced by the fibres to the cell (Nishimura and Broaddus, 1998) during the so-called “oxidative burst” accompanying frustrated phagocytosis. It was observed that the development of 8-hydroxy-2' -deoxyguanosine (8-OHdG) formation by asbestos fibres could be iron-dependent through mobilization of intracellular iron or iron sources other than the asbestos fibres *per se* (Fung et al., 1997). For these reasons, the characteristics of the fibre prompting major adverse effects related to the production of ROS (genotoxicity, alteration of DNA repair or genomic instability and epigenetic changes), are also involved in the effects causing the oxidative burst (Table 3).

Chronic inflammation (6.) and immunosuppression (7.) are assumed to be related to the same fibre parameters. In recent immunosuppression paradigms (Huaux, 2018, Park et al., 2018), asbestos, nanoparticles and other exotic particles found in the lungs promote strongly linked innate (inflammation) and adaptive responses (immunosuppression) that play both a synergic and antagonistic action leading to the onset of cancer (Ngobili and Daniele, 2016). The interplay of inflammation and immunosuppression is witnessed by the fact that during the pro-inflammatory activity, an overexpression of immunosuppressive mediators (cytokines TGF-β and IL-10) mediated by T limphocytes, M2-polarized macrophages and accumulation of potent immunosuppressive MDSC is observed (Huaux, 2018). The assumption that the fibre parameters active for chronic inflammation are also active for immunosuppression is corroborated by the observations that the physical–chemical properties of nanoparticles promoting immunosuppression (namely particle size and shape, surface charge and activity, iron and metal content, metal oxidation state are the same observed for mineral fibres (Ngobili and Daniele, 2016).

Modulation of receptor-mediated effects (8.), alteration of the routes of cell cycle (proliferation and differentiation) and signalling pathways (10.) are key characteristics of cancer that are strongly correlated and both perturbed by mineral fibres. Iron-rich amphibole asbestos fibres (Zanella et al., 1996, Zanella et al., 1999, Manning et al., 2002, Shukla et al., 2003a, Shukla et al., 2003b, Liu et al., 2010) show a direct interaction of their surface with cell-surface receptors inducing protein aggregation and phosphorylation (e.g.*,* protein kinase Cδ-dependent modulation of extracellular signal-regulated kinase ERK1/2 and c-Jun N-terminal kinase (JNK) 1/2 phosphorylation and Bim-associated intrinsic apoptosis in lung epithelial cells). Alteration of cell cycle is also due to the interaction of the fibres with cell surface receptors and production of ROS/RNS, activating a cascade of different cellular signalling pathways such as interleukin 8 (IL-8), fibroblast growth factors (FGFs), MAPK, TNF-α, NF-κβ and many more) and p53 expression (Janssen et al., 1995, Kamp, 2009, Liu et al., 2010, Mossman and Gualtieri, 2020). Activation of RTK (receptor tyrosine kinases), ERK1/2 and phosphatidyl 3-kinase-kinase/AKT pathways have been observed after exposure to asbestos fibres (Chen et al., 2010, Mossman and Gualtieri, 2020). Specifically, crocidolite induces increases in unphosphorylated and phosphorylated ERK1 and ERK2 in bronchial and alveolar type II epithelial cells (Mossman and Gualtieri, 2020) and mesothelial cells (Zanella et al., 1996, Zanella et al., 1999). The characteristics of mineral fibres prompting major effects that may induce modulation of receptor-mediated effects (8.) and alteration of cell cycle and signalling pathways (10.) are related to the surface properties (Kim et al., 2016) and chemistry (Table 3). Cell signalling pathways can be affected by the cation exchange properties exhibited by zeolite mineral fibres like erionite (Carbone and Yang, 2012). Cation exchange may bias the calcium, sodium and potassium mediated intracellular signalling pathways and cross talk because these cations can be exchanged inside the zeolite micropores *in vivo*.

A closer inspection of Table 3 emphasizes the actual importance of fibre parameters in determining cancer-related adverse effects. One of the most significant parameters is the length (1,1) of a fibre (as postulated by the “fibre toxicity paradigm”: Poland et al., 2009) as opposed to the width (1,2). This proviso is not universally shared. For example, animal experiments using anthophyllite and tremolite fibres suggest that the fibre diameter (width) is a more critical factor than the fibre length in the asbestos-induced carcinogenic process by intraperitoneal administration (Aierken et al., 2014, Okazaki et al., 2020). Nevertheless, width is of paramount importance to indicate if a fibre is respirable (when the width < 3 µm and length/width (aspect) ratio > 3) and whether it will reach the alveolar space and pleural/peritoneal surface (Wylie et al., 2020 and references therein). However, length governs the biological interactions and frustrated phagocytosis with all related adverse effects leading to cancer hallmarks (genotoxicity, alteration of DNA repair, epigenetic alteration, oxidative stress, chronic inflammation …).

Besides fibre morphology, another property of the “fibre toxicity paradigm” (Poland et al., 2009, Gualtieri et al., 2017) is biodurability, identified by the dissolution rate (1,12) in the FPTI model. According to the “fibre toxicity paradigm”, the toxicity of a fibre can be assessed to a first approximation by its morphometry and biodurability, assuming that long, thin and biodurable fibres are highly toxic. The parameters of the FPTI model confirm that length and biodurability related parameters (including (1,13), (1,14) and (1,15)) play a key role in determining toxicity/pathogenicity as they cause adverse effects linked to nearly all key characteristics of cancer. However, there are other physical and chemical parameters equivalent to size and biodurability linked to a number of key hallmarks of cancer. Among them, the surface area (1,7), the chemical parameters related to the content of metals (1,8), (1,9), (1,10), (1,11), and the zeta potential (1,16) are also linked to nearly all key characteristics of cancer. The “fibre toxicity paradigm” turns out to be only an approximate model. It is the nature of the mineral fibre in its entirety that dictates the toxic and pathogenic action *in vivo* and therefore its power to induce cancer.

It can also be observed that apparently less relevance is played by the curvature of the crystal lattice of the fibre (whether it is cylindrical or not) (1,3), the aggregation of the fibres (1,17) and cation exchange (1,18) as they cause adverse effects linked to few key characteristics of cancer.

Crystal habit (1,4) and fibre density (1,5) are physical parameters that do not have a direct connection with the key characteristics of cancer. It should be remarked that crystal habit (whether a fibre possesses a curly or needle-like crystal shape) determines the deposition depth of the fibres in lung tissues and does have an indirect influence on carcinogenicity. Mossman et al. (1990) reported that limited alveolar penetration of curly chrysotile bundles may account for the apparent lack of association with the development of MM in human cohorts. On the other hand, if a fibre bundle reaches the lower respiratory tract, it persists at site of deposition and eventually triggers an inflammatory mechanism linked to high mobility group protein B1 (HMGB1) secretion (Carbone et al., 2019) and inflammasome activation (Dostert et al., 2008). Fibre density is also an important parameter as it determines the value of the fibre aerodynamic diameter (D_ae_) responsible for the deposition depth of the fibre in the respiratory tract, with a deeper deposition depth (deep respiratory system) for denser fibres (*i.e.* fibres with larger D_ae_) (Gualtieri et al., 2017).

A final comment should be added on the dissolution of silica (parameter (1,14) in Table 1). The formation of a silica-rich fibre skeleton after amorphization of mineral fibres (namely chrysotile), characterized by silanol groups (Si-OH) and ionized silanol groups (Si-O^-^), may prompt production of HO•, in synergy with surface iron species (Pollastri et al., 2014). ROS formation in both silica phases like quartz (Pavan et al., 2019) and silica nanophases (Lehman et al., 2016) is a very active research field. Although the mechanism for silicates other than quartz is not well understood (Gualtieri et al., 2019c), the formation of a reactive silica surface and its subsequent dissolution should be considered as possible source of ROS.

## 4 Not all mineral fibres are equal. Chrysotile vs. Crocidolite

Table 4 reports the values of the FPTI model calculated for standard UICC (Union for International Cancer Control) chrysotile and crocidolite (Gualtieri, 2018). The different final FPTI (2.22(0.28) for chrysotile and 2.73(0.18) for crocidolite, respectively) reflects a different overall toxicity/pathogenicity potential for these fibres that is the result of the different weights of single or group parameters for the two species. In more detail, some fibre parameters (length, width, surface hydrophobic character, ferrous iron and its nuclearity) of the two mineral fibres yield identical FPTI_i_ scores; other fibre parameters like surface area, total iron content, content of metals other than iron, velocity of iron and metals release, and aggregation of the fibres yield comparable FPTI_i_ scores; only a few parameters (crystal curvature, biodurability in terms of dissolution rate, velocity of silica release) of the two fibre species have significantly different FPTI_i_ scores and should be responsible for their diverse behaviour *in vivo*.

**Table 4:** The calculated FPTI for the UICC standard chrysotile “B” asbestos, UICC standard crocidolite (NB #4173-111-3) and fibrous glaucophane, Marin County, Franciscan Complex (CA, USA). FPTI of the mineral fibres has been computed on April 2020 using the WebFPTI application available at *fibers-fpti.unimore.it*.

Parameter	classes	Normalized score FPTI_i_	UICC chrysotile	UICC crocidolite	Fibrous glaucophane (CA, USA)
(1,1)	>5μm and <10 μm >10 μm and <20 μm >20 μm	0.10 0.20 0.40	0.40	0.40	0.00 (<5μm)
(1,2)	>1μm and <3 μm >0.25 μm and <1 μm <0.25 μm	0.10 0.20 0.40	0.20	0.20	0.40
(1,3)	Flat surface (perfect crystal) Altered surface Cylindrical surface	0.05 0.10 0.20	0.20	0.05	0.05
(1,4)	Curled Mixed Curled/acicular Acicular	0.10 0.20 0.40	0.10	0.40	0.40
(1,5)	<2.75 g/cm^3^ >2.75 and <3.5 g/cm^3^ >3.5 g/cm^3^	0.05 0.10 0.20	0.05	0.10	0.10
(1,6)	Hydrophobic Amphiphilic Hydrophilic	0.05 0.10 0.20	0.20	0.20	0.20
(1,7)	>25 m^2^/g <25 and >5 m^2^/g <5 m^2^/g	0.05 0.10 0.20	0.05	0.10	0.10
(1,8)	Fe_2_O_3_ + FeO wt% <1 1 < Fe_2_O_3_ + FeO wt% < 10 Fe_2_O_3_ + FeO wt% >10	0.05 0.10 0.20	0.10	0.20	0.20
(1,9) ferrous iron	0 < FeO wt% <0.25 0.25 < Fe Owt% <1 FeOwt% >1	0.05 0.1 0.2	0.20	0.20	0.20
(1,10)	Fe^2+^ nuclearity > 2 Fe^2+^ nuclearity = 2 Fe^2+^ nuclearity = 1	0.02 0.03 0.07	0.03	0.03	0.02
(1,11)*	$\sum_{\text{i}}\frac{{\text{C}}_{\text{i}}}{{\text{L}}_{\text{i}}}$ < 1 1 < $\sum_{\text{i}}\frac{{\text{C}}_{\text{i}}}{{\text{L}}_{\text{i}}}$< 5 $\sum_{\text{i}}\frac{{\text{C}}_{\text{i}}}{{\text{L}}_{\text{i}}}$ > 5	0.10 0.20 0.40	0.20	0.20	0.40
(1,12)℘	<1y >1 and < 40y >40y	0.05 0.1 0.2	0.05	0.20	0.20
(1,13)ℵ	<0.1 >0.1 and <1 >1	0.03 0.07 0.13	0.13	0.07	0.07
(1,14)ℑ	<0.5 >0.5 and <1 >1	0.02 0.03 0.07	0.07	0.02	0.07
(1,15)^R^	<1 >1 and <10 >10	0.03 0.07 0.13	0.07	0.13	0.13
(1,16)	Negative at pH = 4.5 Negative at pH = 4.5 and 7	0.1 0.2	0.10	0.20	0.20
(1,17)	>|20| |10|< and <|20| |0|< and <|10|	0.03 0.07 0.13	0.07	0.03	0.03
(1,18)	cation Exchange no cation exchange	0.07 0	0	0	0
		FPTI (error)	2.22(0.28)	2.73(0.18)	2.77(0.25)

* $\sum_{\text{i}}\frac{{\text{C}}_{\text{i}}}{{\text{L}}_{\text{i}}}$ = sum of the concentrations of heavy metals (Sb, As, Hg, Cd, Co, Cr, Cu, Pb, Ni, Zn, V, Be) C_i_ in the fibre (ppm) divided by the limit L_i_ for that metal according to the existing regulatory system (Gualtieri, 2018) except for Be with limit = 0.5 ppm.

℘ the total dissolution time of the fibre calculated in years (y) following the standardized acellular *in vitro* dissolution model at pH = 4.5 described in Gualtieri (2018).

ℵ total content of elemental iron in the fibre (wt%) possibly made available as active iron at the surface of the fibre divided by the total dissolution time (y) of the fibre (y).

ℑ total content of Si of the fibre (wt%) divided by the total dissolution time (y) of the fibre.

R total content (ppm) of heavy metals (Sb, As, Hg, Cd, Co, Cr, Cu, Pb, Ni, Zn, V, Be; Mn, Be) divided by the total dissolution time (y) of the fibre.

Concerning the crystal curvature (1,3), chrysotile has a cylindrical lattice with a curved surface whereas crocidolite has a flat crystal surface. Hence, at the same surface area and hydrophobicity, protein interaction and adsorption onto the chrysotile surface are not favoured while they occur on the crocidolite surface because protein adsorption on the curved surface can be suppressed up to the point when it no longer occurs (Deng et al., 2012). Besides specific surface and surface chemistry, crystal curvature can explain why crocidolite and amosite fibres yield similar protein adsorption profiles whereas that of chrysotile was distinct and why much less protein was adsorbed on silica than asbestos (Nagai et al., 2011). In this regard, for silica nanoparticles it was observed that those particles with a longer diameter (lower surface curvature) allowed formation of larger particle-protein interaction surfaces and caused larger perturbations of the protein's secondary structure upon interaction (Lundqvist et al., 2004).

The key parameter that distinguishes chrysotile from crocidolite is the dissolution rate (1,12), the key biodurability factor. Chrysotile is not biodurable while crocidolite is (Jaurand et al., 1977, Bernstein et al., 2013, Gualtieri et al., 2018a). Dissolution rate determines a time-dependent cascade of adverse effects produced by a mineral fibre like the indirect production of ROS, production of ROS/RNS during AM frustrated phagocytosis, the persistence at site of deposition, triggering chronic inflammatory activity and these adverse effects are responsible for most of the key characteristics of carcinogenicity from electrophilicity (1.) to immunosuppression (7.) (Table 3). Hence, non-biodurable chrysotile is less active in stimulating all those key characteristics of carcinogenicity than crocidolite.

The rate of silica release/formation (1,14) is another distinctive parameter between chrysotile and crocidolite. As it is not biodurable, silica dissolution and release are much faster in chrysotile with respect to biodurable crocidolite. Silica formation (the relicts of chrysotile) may prompt production of genotoxic ROS/RNS *in vivo* (Gualtieri et al., 2019c) eventually responsible for electrophilicity (1.), genotoxicity (2.) and oxidative stress (5.) key characteristics of cancer (Table 3). In this case, non-biodurable chrysotile is more active than crocidolite in triggering all these key characteristics of carcinogenicity.

## 5 Implications

An implication of the model presented in this work is the possibility to classify newly-discovered unregulated mineral fibres in order to assess *a priori* if they are potentially toxic/pathogenic and should be recommended for *in vitro*/*in vivo* toxicity testing. The natural environment contains several mineral fibres sharing the same characteristics of asbestos and possibly its potential adverse health effects (Carbone et al., 2007, Naik et al., 2017). For example, the risk posed by unregulated fibrous amphiboles (e.g., winchite and richterite) in Libby, Montana has been proven by several studies and, although they have not yet been classified by the IARC, specific *in vitro* and *in vivo* tests point out that they represent a health potential hazard (Baumann et al., 2015, Naik et al., 2017).

An example of unclassified unregulated mineral fibre is fibrous glaucophane from California, USA, whose FPTI data (after Di Giuseppe et al., 2019) are also reported in Table 4. Glaucophane is an alkaline amphibole with ideal chemical formula Na_2_[(Mg,Fe^2+^)_3_(Al,Fe^+3^)_2_]Si_8_O_22_(OH)_2_. Erskine and Bailey (2018) recently found that glaucophane can assume a fibrous habit resembling amphibole asbestos. It occurs in blueschist facies such as at the Franciscan Complex, exposed from the California-Oregon border to Los Angeles, USA (Erskine and Bailey, 2018). Blueschist rocks from Franciscan Complex are commonly mined for building/construction purpose in northern and central California (e.g., Calaveras Dam Replacement Project – CDRP; Erskine and Bailey, 2018) and the dust generated by the excavation activities may potentially expose workers and the nearby populations to adverse health risks. For this reason, fibrous glaucophane may represent a health hazard as naturally occurring asbestos (NOA) and an evaluation of the toxicity/pathogenicity potential of this mineral fibre is highly recommended. The FPTI of fibrous glaucophane collected at San Anselmo, Marin County (CA, USA) is comparable to that of crocidolite (2.77(0.25) vs. 2.73(0.18) in Table 4). Among the parameters active in stimulating key characteristics of carcinogenicity, most of them are undistinguished or comparable between the two species (width, crystal habit, surface hydrophobicity, total and ferrous iron content, content of metals, dissolution rate, velocity of release of metals, zeta potential). Only the length and the silica dissolution rate display significantly different FPTI values. The mean length of the fibrous glaucophane fibres of the investigated sample from San Anselmo, Marin County (CA, USA) is < 5 μm (Di Giuseppe et al., 2019) and hence this parameter is inactive in stimulating all the related key characteristics of carcinogenicity that long crocidolite fibres do. On the other hand, the glaucophane fibres are very thin (50% of the fibres display a width <0.22 µm: Di Giuseppe et al., 2019) and this is a source of concern as thin fibres enter the deep lung system. Surprisingly, Wylie et al. (2020) found that there are few (about 1%) fibrous glaucophane fibres from Calaveras dam (CA, USA) of the width that will enter the deep lung and be transported to the mesothelial surface.

Concerning the other parameters, the velocity of silica dissolution is greater in fibrous glaucophane. Dissolution eventually prompts the production of ROS (namely HO•) from the newly-formed silica rich layer at the fibre surface (Fenoglio et al., 2001) which in turn stimulates the cancer key characteristics electrophilicity (1.), genotoxicity (2.) and oxidative stress (5.). As explained in the introduction of this paper, indirect production of electrophilic species like HO•, with strong electrophilic character, are able to attack a great variety of target molecules (Zalma et al., 1987) *in vivo* and promote the formation of oxidized DNA base products, such as hydroxy-2′-deoxyguanosine, resulting in mutations and development of cancer (Cooke et al., 2003).

Another fascinating implication of the model presented here is that identifying the fibre parameters that stimulate specific key characteristics of cancer can help to provide targeted prevention strategies and therapies, given that the nature of the fibre the patient was exposed to is assessed by microscopic examination of statistically significant broncho-alveolar lavage samples or other methods. Along this line, Wang et al. (2016) recently investigated the role of specific protein adsorption on the surface of carbon nanotubes in causing mesothelial iron overload and contributing to oxidative damage and possibly subsequent carcinogenesis in mesothelial cells and postulated that modifications of the surface may decrease this human risk. Although not yet endorsed by clinicians, another possible specific therapy in MM is to decrease the iron stores on the surface of the asbestos fibres either by redox-inactive iron chelators or phlebotomy (Toyokuni, 2013).

In the introduction of this paper it was said that the developed model provides a basic quantitative paradigm to predict the toxicity/pathogenicity potential of mineral fibres hosted in the lung environment. In the future, if widely accepted and considered a solid basis for toxicologists, the model should be merged with other existing models to incorporate toxicity dose/time dependency and cancer dose–response relationship.

## 6 Concluding remarks

Characterization of mineral fibres by geologists is central to better understanding their effects on health, with special attention to the risk for development of MM and asbestos associated diseases. Along that research line, this work is the outcome of a long-term project on the bio-chemical interaction of mineral fibres *in vivo* and delivers an attempt to create an innovative approach which correlates the physical/crystal-chemical and morphological parameters of a mineral fibre to the major adverse effect they cause *in vivo* responsible for the patho-biological processes classified as key characteristics of cancer. The perspective of a mineralogist/chemist that drives the project can be different from that of a pathologist or a geneticist who believe that biological responses are more important to assess the human risk of mineral fibres in addition to the characteristics of the fibres themselves but it is of paramount importance to give an attempt to reconcile these different worlds as a multidisciplinary approach is the only key to disclosing the very mechanisms of asbestos-induced carcinogenesis (Gualtieri, 2017b).

The author is aware that, at the moment, the predictive power and the impact of the model are limited as only few cases have been considered so far. The planned roadmap is to create a database of mineral fibres whose mineralogical/chemical/physical properties and toxicity/pathogenicity effects are fully assessed and interpreted in terms of carcinogenicity. To do this, for each fibre, the first step is to assess the toxicity/pathogenicity potential using the FPTI model (taking advantage of the available WebFPTI application available at *fibers-fpti.unimore.it*) and verify the prediction with *in vitro* toxicity tests and eventually *in vivo* testing. For some fibres such data are already available from the literature. As starting case study, we have focussed on fibrous glaucophane which represents a great concern in California (USA). The analysis of fibrous glaucophane from San Anselmo, Marin County (CA, USA) shows that its toxicity/pathogenicity potential is comparable to that of crocidolite, the only major differences being the fibre length and the silica dissolution rate. With respect to crocidolite, the short but thin glaucophane fibres (Di Giuseppe et al., 2019) do not stimulate the key characteristics of carcinogenicity like long thin crocidolite fibres do. On the other hand, the greater velocity of silica dissolution in fibrous glaucophane can be responsible for the cancer key characteristics electrophilicity (1.), genotoxicity (2.) and oxidative stress (5.).

Regarding the standard UICC chrysotile and crocidolite reference samples, they exhibit different overall toxicity/pathogenicity potential due to the different action of parameters like the crystal curvature, biodurability, velocity of silica release, and zeta potential. These parameters are responsible for the distinct behaviour of the two fibres *in vivo*. Specifically, chrysotile has a cylindrical lattice and protein interaction is not favoured onto its surface (Deng et al., 2012) causing the stimulation of both modulation of receptor-mediated effects (8.) and alteration of cell cycle/growth factors and signalling pathways (10.). The major difference between chrysotile and crocidolite is the dissolution rate that determines a cascade of adverse effects in crocidolite with respect to chrysotile responsible for most of the key characteristics of carcinogenicity. As chrysotile is not biodurable, silica dissolution and release are much faster with respect to crocidolite and may cause adverse effects responsible for electrophilicity (1.), genotoxicity (2.) and oxidative stress (5.). The zeta potential is also a key distinctive feature and is linked to key characteristics of cancer from genotoxicity (2.) to immunosuppression (7.).

## CRediT authorship contribution statement

**Alessandro F. Gualtieri:** Conceptualization, Methodology, Writing - original draft, Writing - review & editing.

## Declaration of Competing Interest

The author declares that they have no known competing financial interests or personal relationships that could have appeared to influence the work reported in this paper.
